# A process-based life cycle assessment of the climate impact of a Swedish intensive care unit

**DOI:** 10.1038/s41598-025-02789-z

**Published:** 2025-06-03

**Authors:** Linn Hemberg, Jagdeep Singh, Peter Bentzer

**Affiliations:** 1 Anaesthesia and Intensive Care, Department of Clinical Sciences Lund, Lund University, Lund, Sweden; 2 Lund University Agenda 2030 Graduate School, Lund University, Lund, Sweden; 3 Centre for Environmental and Climate Science, Faculty of Science, Lund University, Lund, Sweden; 4 Department of Anaesthesia and Intensive Care, Helsingborg Hospital, Helsingborg, Sweden

**Keywords:** Climate impact, Carbon dioxide, Intensive care, Critical care, Life cycle assessment, Mitigation strategies, Health care, Environmental impact

## Abstract

About 4.4% of the global emissions of greenhouse gases can be ascribed to healthcare. Intensive care is among the most resource intensive specialties and the purpose of this study was to quantify the total climate impact of a Swedish intensive care unit (ICU) and identify modifiable elements that can lower the intensive care unit’s climate impact. A process-based life cycle assessment was conducted to estimate the climate impact per inpatient day in Sweden using the ReCiPe2016 impact assessment method. The analysis included single-use items; reusable instruments and textiles; pharmaceuticals and fluids; medical gases; and energy consumption for electronics equipment, lighting, and heating, ventilation, and air conditioning (HVAC). Input data were collected in 2022 at a mixed surgical and medical ICU in Sweden. Swedish low-climate-impact energy mix were used in the primary analysis. Results are reported as the global warming potential of carbon dioxide equivalents for 100 years (CO_2_eq). The median climate impact of one inpatient day was 30 kg CO_2_eq (95%-reference interval: [27–31]). Approximately 63% (19 kg CO_2_eq [18-20] could be attributed to single-use items, 19% (5.5 kg CO_2_eq [4.3–7.9]) was attributed to the unit’s energy consumption, pharmaceuticals and fluids contributed 7% (1.9 kg CO_2_eq [1.7–2.2]), and 5% (1.5 kg CO_2_eq [1.2–1.9]) was attributed to medical gases. A sensitivity analysis, using a high-climate-impact energy mix increased the total climate impact to 126.5 kg CO_2_eq (103–154). In countries with low-climate-impact energy mixes, such as Sweden, the opportunity to reduce the climate impact of intensive case lies primarily in the reduced use of single-use items. For countries that depend on high-climate-impact energy mixes, the foremost opportunity to reduce the climate impact of intensive care is to transition to renewable energy.

**Supplementary Information:**

The online version contains supplementary material available at 10.1038/s41598-025-02789-z.

## Introduction

Climate change already impacts human health and is perceived as the most significant health issue of the 21st century^1-3^. Roughly 4% of the global emissions of greenhouse gases can be ascribed to the health sector^4^, necessitating the identification of mitigation strategies. The production, transport, use, and waste management of goods and services account for about 70% of the healthcare’s total emissions, and healthcare facilities’ energy use from self-produced or purchased electricity, steam, cooling, and heating account for about 30%^4^.

Several studies have assessed the impact of specific instruments or processes that are used in the intensive care unit (ICU), including instruments and textiles for central venous catheters^5,6^, infusion sets^7^, instruments for laboratory measurements^8^, and pathology testing^9^. In a study that estimated the impact of disposable items used at an ICU in the Netherlands, five environmental hotspots were identified: non-sterile gloves, isolation gowns, bed liners, surgical masks, and syringes^10^. However, less is known about the overall climate impact of intensive care and the relative contribution of various sources of emissions^11^. This information is essential when prioritising interventions proposed to reduce intensive care’s climate impact. The climate impact of treating patients with septic shock in ICUs in Australia and the US has been estimated in an analysis that included consumables, pharmaceuticals, radiology tests, pathology tests, electricity, and energy for heating, ventilation, and air conditioning (HVAC)^12^. The study found that the major source of this impact was the energy used for HVAC. In contrast, the major climate impact of a medical ICU in the US could be referred to consumables^13^. Both studies used a hybrid life cycle assessment (LCA) approach, combining an economic input-output LCA (EIO-LCA) that estimated climate impact per dollar spent with a process-based LCA that measured the climate impact of the materials and processes used within a system. EIO-LCAs are sensitive to price fluctuations due to an assumed linear relationship between the financial cost and the emissions of products and services within specific economic sectors. Due to this sensitivity and the contrasting results of previous studies, a completely process-based LCA has been proposed to assess the climate impact of intensive care^13^.

Based on the findings above, this study addresses the need for an exclusively process-based LCA to enable a more detailed assessment of the impacts of intensive care. The study’s primary objective was to estimate the climate impact per inpatient day of treating patients in an ICU and to identify modifiable elements. A secondary aim was to assess the external validity of our conclusions in a sensitivity analysis using an alternate energy mix.

## Method

LCA is a quantitative method that can be used to estimate the environmental impact of a process or product, including all impacts, from raw material extraction to waste management, in the analysis. The principles and framework of LCA are described in International Organization for Standardization (ISO) 14040:2006 and ISO 14044:2006^14–15^. Thus, the study was performed according to the following four steps: (i) defining the goal and scope, (ii) inventorying material and process inputs and outputs, (iii) assessing the impact, and (iv) interpretating the results, including those of sensitivity and uncertainty analyses. Specific assumptions considered in each step are described below.

### Goal and scope

**Fig. 1:**
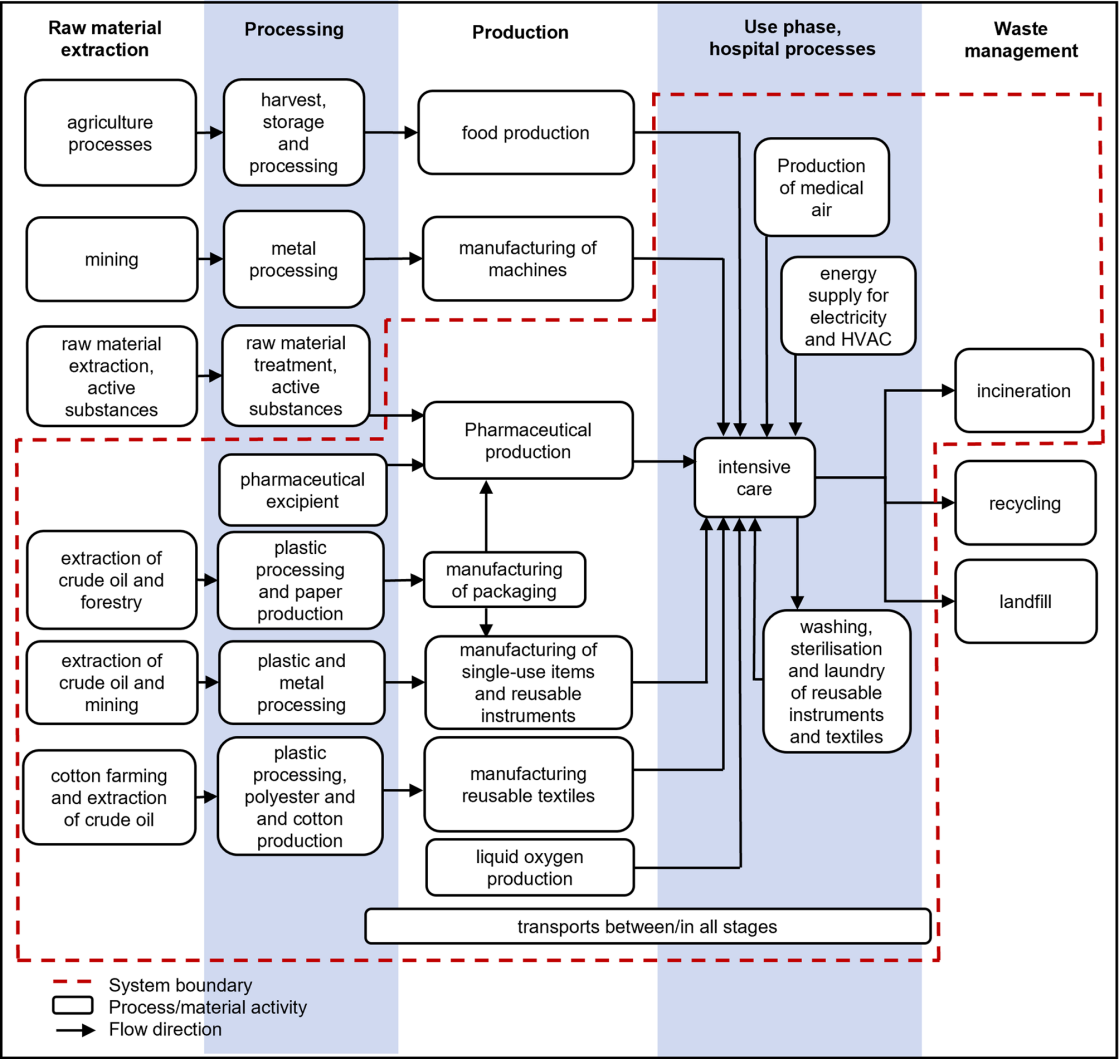
System boundaries of included items. system boundaries of included items (within the dotted lines) in this life cycle assessment.* HVAC*  heat, ventilation, and air conditioning.

This study aimed to estimate the climate impact per inpatient day of an ICU in Sweden. Additionally, the study aimed to identify modifiable elements that healthcare professionals and administrative staff can target to reduce the climate impact of intensive care. This ICU cares primarily for adult medical and surgical patients and has 4 treatment rooms with 8 beds. The analysis included single-use items, reusable instruments and textiles, pharmaceuticals and fluids, medical gases, and energy consumption for HVAC, electronic equipment, and lighting. The manufacturing of capital goods was excluded from the analysis since the allocated contribution was assumed to be negligible^16^. Patient food services were excluded from the analysis since ICU patients in Sweden rarely eat and instead receive enteral or intravenous nutrition. Active Pharmaceutical Ingredients (APIs) were excluded from the primary analysis because they are complex and laborious to model. See Fig. 1. for system boundaries.

### Inventory

We used an attributional, process-based LCA approach to create an ICU model, based on physical material and process flows. The model was created using the LCA software SimaPro v.9.2.0.2. Material and process data were sourced from the ecoinvent 3.6 database^17^. Only the primary material and connected processes of each item or service were included in the analysis. Thus, if a product’s weight could be attributed primarily to a plastic, we included only that plastic in our inventory. The weight of all products was measured on site at the hospital on a KERN KB-N, KB 6500–1 N balance, with a precision of ± 0.1 g. Waste generated at the ICU is generally treated as hazardous waste and incinerated and all waste management was therefore modelled as municipal incineration.

Input data were collected from January 1 to December 31, 2022, from the ICU at Helsingborg Hospital in southern Sweden. Information on patient demographics was retrieved from the Swedish Intensive Care Registry^18^. Quantities of single-use items, reusable instruments, and pharmaceuticals and fluids were retrieved from procurement records. Information on materials and processes that were used to manufacture these items was collected from the manufacturers’ or distributors’ websites. For items without such information, the materials and production processes were assumed to be identical to those for similar products. Data needed to model the sterilisation process of reusable items was provided by the sterile technical unit at the hospital. Quantities of reusable textiles were based on data from the unit’s administrative records and physical inventory in the unit’s storage. Data needed to model the production of textiles as well as the laundry process was provided by the hospital’s textile and laundry service. Inventory of medical gas consumption was based on the number of hours of each included respiratory support mode^18^, and data needed to model the production of medical gases was provided by the hospital’s engineers. Data on the unit’s energy consumption for electricity and HVAC was also provided by hospital engineers. The energy consumption for electronic equipment and lighting was modelled with an average Swedish electricity mix (40% nuclear power, 40% hydro power, 10% wind power, and 10% thermal power^19^). The same electricity mix was used to model the sterilisation and laundry process of reusable textiles and instruments. The energy requirement for HVAC was modelled as Swedish cogeneration with biogas (biowaste, sewage sludge). The type and number of diagnostic imaging procedures were retrieved from the unit’s administrative records, and the data needed to model diagnostic imaging were based on a previous study^20^, adapted to a Swedish setting. Further details on assumptions concerning each inventory category are provided in Table 1. For detailed information on the inventory for each included category, item, and process, please see Additional file 1.

**Table 1:** Inventory categories included in the analysis and their associated assumptions.

ICU category	Assumptions
Single-use items	Items of which a total of 1000 pieces or more had been ordered during the year were included.
Reusable instruments	The sterilisation of reusable instruments was based on average consumption data for a fully loaded washer-disinfector (*Getinge AB*, model: 46-series) used at the ICU and average consumption data for a fully loaded autoclave (*Getinge AB*, model: HS6617 ER-2) used at the sterile technical unit.
Reusable textiles	The laundry process was based on consumption data regarding water, electricity, and wastewater treatment for 1 kg of textile, provided by the technical advisor at the laundry facility in Kristianstad, Sweden. Lorries were used for transport between the hospital and laundry facility, and we assumed the shortest path for transport using Google Maps.
Pharmaceuticals and fluids	The main excipient and the product packaging were included in the analysis. Products of which a total of 50 or more packages had been ordered during the year were included.
Medical gases	Respiratory support modes for which we could retrieve data were high-flow oxygen therapy and non-invasive and invasive ventilation. Thus, medical air and oxygen were included in the analysis. We modelled high-flow oxygen therapy with a flow of 50 l/min and an FiO_2_ of 50%, non-invasive ventilation with a flow of 15 l/min and an FiO_2_ of 40%, and invasive ventilation with a flow of 10 l/min and FiO_2_ of 40%. Data for oxygen therapy using nasal prongs or oxygen masks could not be retrieved.
Electricity and HVAC	The energy consumption for electronic equipment and lighting as well as HVAC was modelled as energy consumption (kWh) per m^2^ and year.
Diagnostic imaging	The climate impact of diagnostic imaging on the ICU’s total climate impact was assessed by adapting the model from a previous process-based LCA [15] to a Swedish setting. Briefly, for magnetic resonance imaging (MRI), computed tomography scan (CT), and ultrasound imaging, we used the Swedish electricity mix described above and assumed the same impact as the previous study for consumables. We assumed the same climate impact of X-rays as reported, because nearly all impact was represented by consumables [15]. Detailed information on the adaptation is available in Additional file 1.

### Life cycle impact assessment

Climate impact was estimated using the ReCiPe2016 impact assessment method and expressed as the global warming potential of carbon dioxide equivalents over 100 years (GWP_100_ CO_2_eq)^21^ and is presented as kg CO_2_eq per inpatient day (24 h).

### Sensitivity analyses

Many countries use energy mixes with a higher climate impact than Sweden and to assess the impact of the energy mix on our results, we performed a sensitivity analysis in which we used a high-climate-impact energy mix. For this purpose, we used a Polish electricity mix to model the unit’s electricity consumption for electronic equipment and lighting, liquid oxygen production, diagnostic imaging, and the sterilisation and laundry of reusable textiles and instruments. The Polish mix comprised 76% hard coal and lignite, 14% renewable, 8% thermal gas, and 2% nuclear^17^. We used Polish cogeneration with hard coal to model the energy requirement for HVAC in this analysis.

Because the climate impact of pharmaceuticals that are used in the ICU is complex, laborious to model, and rarely known, we excluded them from our primary analysis. To assess the effects of this omission, we performed a sensitivity analysis using recently published data on the climate impact of commonly used active pharmaceutical ingredients (APIs)^22^.

### Statistics and uncertainty

We performed Monte Carlo simulations (*n* = 1000) to assess the uncertainty of the results as detailed previously^23^, and the results are presented as medians with 95% reference intervals.

## Results

### Demographics

In 2022, 539 patients were treated at the ICU in Helsingborg for 1779 inpatient days. Thus, 0.056% (1/1779) of all items and resources that were included in the analysis were allocated to one inpatient day. Patient characteristics and treatment intensity are presented in Table 2.

**Table 2:** Treatment intensity at the ICU at Helsingborg hospital, Sweden for 2022.

Number of admissions	539
Number of patients	495
Number of inpatient days	1779
Postoperative patients	66 (12.2%)
Length of ICU stay in hours	34 (15–87)
Number of patients receiving invasive ventilation	270 (50%)
Total time with invasive ventilation (h)	22,506
Number of patients receiving non-invasive ventilation	101 (19%)
Total time with non-invasive ventilation (h)	2421
Number of patients receiving high-flow oxygen	108 (20%)
Total time with high-flow oxygen (h)	10,248
Number of patients receiving CRRT	41 (8%)
Total time with CRRT (h)	3679

*CRRT*  continuous renal replacement therapy. Data are presented as median (Q1–Q3) or n (%) as appropriate.

### Inventory

A total of 98 types of single-use items and 13 types of reusable instruments were included in the analysis. A total of 0.6 washer-disinfector runs in the ICU were attributed to each inpatient day to wash reusable instruments. A total of 0.09 washer-disinfector runs and 0.009 autoclave runs in the sterilisation unit were attributed to each inpatient day. A total of 10 types of reusable textiles were included in the analysis, and 5.4 kg of textiles was attributed to each inpatient day. The laundry of textiles required 43 l of water and wastewater treatment, 111 km of transport, and 7.1 kWh of electricity per inpatient day. A total of 45 pharmaceutical products and fluids were included in the analysis. The main excipient in most products was ultrapure water.

Medical air was produced on site at the hospital, and liquid oxygen was produced off site in Sweden and delivered to the hospital by lorry. An average of 5.8 h of high-flow oxygen therapy (8641 l of medical air, 8641 l of oxygen), 1.4 h of non-invasive ventilator treatment (735 l of medical air, 490 l of oxygen), and 12.6 h of invasive ventilator treatment (4554 l of medical air, 3036 l of oxygen) were attributed to each inpatient day.

The ICU’s HVAC consumed an average of 74.1 kWh per inpatient day, and electronic equipment and lighting consumed an average of 48.6 kWh per inpatient day. An average of 0.12 CTs, 0.02 MRIs, 0.51 X-rays, and 0.02 ultrasound examinations were performed by the Department of Radiology per inpatient day. For detailed information on the inventory results, please see Additional file 1.

### Climate impact

**Fig. 2:**
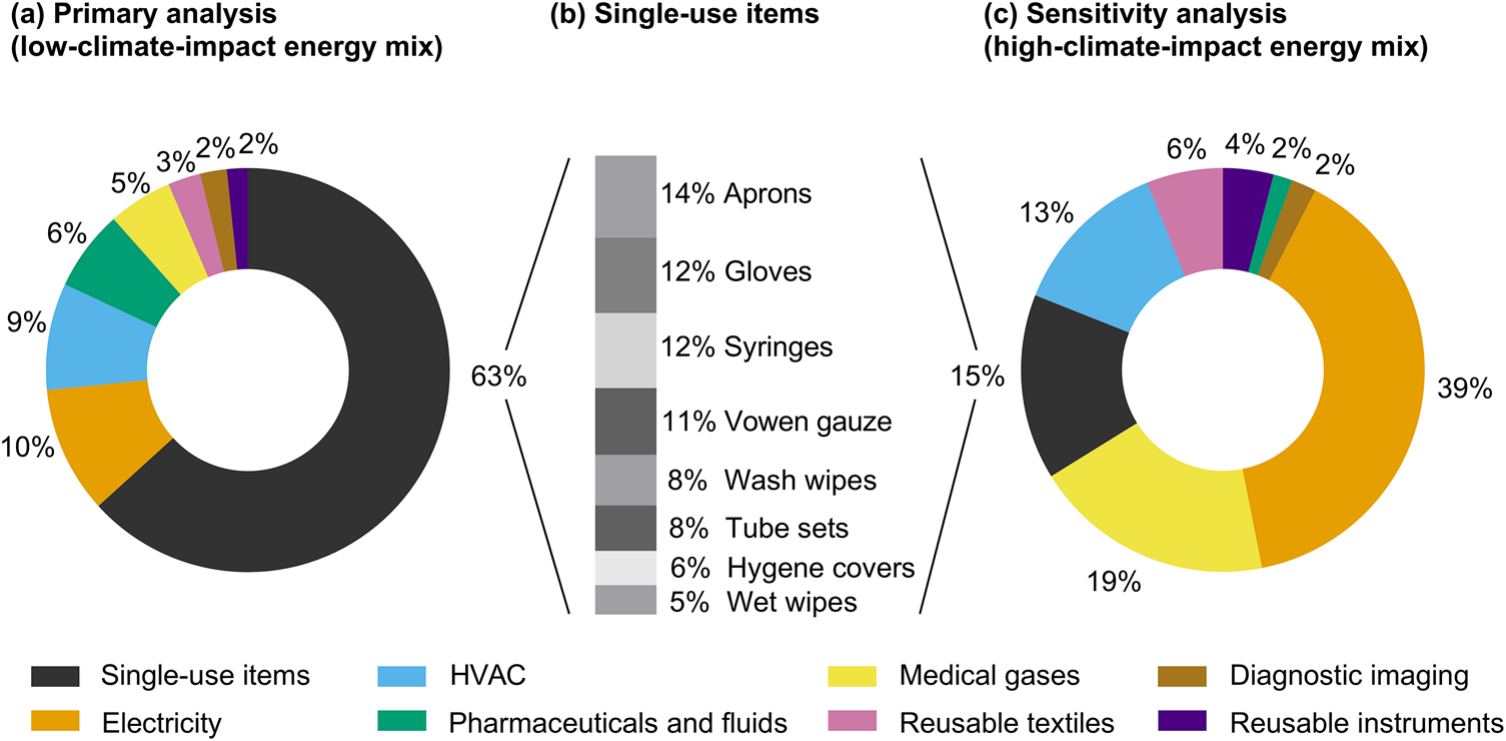
Contribution analysis showing the impact from various activities in the ICU, based on results from the (**a**) primary analysis using a low-climate-impact energy mix, including (**b**) the single-use items that contributed to ≥ 5% of the total impact from single-use items, and (**c**) sensitivity analysis using a high-climate-impact energy mix in the model, including (**b**) the single-use items that contributed to ≥ 5% of the total impact from single-use items.* HVAC * heat, ventilation, and air conditioning.

The median climate impact from one inpatient day was 30 kg CO_2_eq (95%-reference interval: 27–31). The contribution analysis showed that single-use items represented 63% of the total impact, followed by the energy consumption for electric equipment and lighting (10%) and HVAC (9%), pharmaceuticals and fluids (7%), medical gases (5%), reusable textiles (3%), diagnostic imaging (2%), and reusable instruments (2%) (Fig. 2; Table 3). The contribution analysis showed that most of the climate impact of single-use items was generated by 5 items – aprons accounted for 13% of the category’s total climate impact, followed by 12% each for gloves and syringes, 11% for woven gauze, and 8% for wash wipes (Fig. 2). The production of liquid oxygen represented nearly all of the impact from medical gases, of which over 70% was used for high-flow oxygen therapy. Packaging production represented nearly all of the impact of pharmaceuticals and fluids.

**Table 3:** Climate impact per inpatient day for the primary and sensitivity analysis.

Categories	Primary analysis (low-climate-impact energy sources)		Sensitivity analysis (high-climate-impact energy sources)		Increase in climate impact between primary and sensitivity analysis
	kg CO_2_eq per inpatient day	% of total	kg CO_2_eq per inpatient day	% of total	
Single-use items	19 (18–20)	63%	19 (18–20)	15%	-
Electricity	3 (2.3–3.9)	10%	50 (37–67)	39%	+ 1567%
HVAC	2.5 (2–3)	9%	16 (14–20)	13%	+ 540%
Pharmaceuticals and fluids	1.9 (1.7–2.2)	7%	1.9 (1.7–2.2)	2%	-
Medical gases	1.5 (1.2–1.9)	5%	24 (18–33)	19%	+ 1500%
Reusable textiles	0.78 (0.7–0.9)	3%	7.7 (5.7–10)	6%	+ 887%
Diagnostic imaging ^a^	0.63	2%	2.5	2%	+ 297%
Reusable instruments	0.5 (0.4–0.6)	2%	5.1 (3.9–6.8)	4%	+ 920%
**Total** ^**b**^	**30 (27–31)**		**126.5 (103–154)**		**+ 322%**

Primary analysis with low-climate-impact energy mix. Sensitivity analysis with high-climate-impact energy mix. kg CO_2_eq = carbon dioxide equivalent. *HVAC  * heat, ventilation, and air conditioning. Result presented as median (95% reference interval) and percentage of total climate impact. Total climate impact is highlighted in bold font. ^a^ Results adapted from a previous study, and no uncertainty is available^15^. ^b^ Uncertainty range does not cover diagnostic imaging.

### Sensitivity analyses

To assess the impact of the energy mix on our results, we performed a sensitivity analysis in which we replaced the Swedish energy mix with the high-climate-impact energy mix used in Poland. Using a high-impact energy mix increased the climate impact by 322% to a median of 126.5 kg CO_2_eq per inpatient day (Table 3). This increase could primarily be explained by an increased impact from electricity, HVAC, and medical gases.

To assess the effects of not including APIs in our primary analysis, we performed a sensitivity analysis using previously published data on the climate impact of 12 common APIs used in the ICU. The analysis only marginally increased the climate impact per inpatient day, by 0.05 kg CO_2_eq or 0.1% (Table 3).

## Discussion

The principal finding of this study was that one inpatient day at an ICU in Sweden had a climate impact of 30 kg CO_2_eq. Over 60% of the total climate impact could be attributed to using single-use items, followed by energy consumption for electric equipment, lighting, and HVAC (roughly 20%), and medical gases (5%). A sensitivity analysis showed that a high-climate-impact energy mix increased the climate impact by 322% to 126.5 kg CO_2_eq.

Our results suggest that the total climate impact of an inpatient day at a Swedish ICU (30 kg CO_2_eq) is much lower than the 88 kg and 178 kg CO_2_eq that have been reported for septic ICU patients in Australia and the US, respectively, and the 136 CO_2_eq that has been reported for medical ICU patients in the US^12–13^. As suggested by our sensitivity analysis with a high-climate-impact energy mix, the difference in results is explained by the fact that Sweden mainly uses a fossil-free low-climate-impact energy mix. Our sensitivity analysis showed that energy consumption and single-use items contributed approximately 80% and 15% of the total climate impact, respectively, when using a high-climate-impact energy mix, which aligns broadly with that for septic ICU patients who are treated in the US and Australia^12^. In contrast, the relative contribution of energy was lower in a medical ICU in the US, at 30%, and that of single-use items was higher, at roughly 30%^13^. A hybrid LCA approach and the inclusion of electronic equipment, staff travel, and patient food within the system boundaries could explain the differing results of the latter study.

The medical grade oxygen production process is highly energy-intensive. As found by our sensitivity analysis, the climate impact from medical gases increased from 1.5 kg CO_2_eq to 24 kg CO_2_eq when the 12,000 l of oxygen that were attributed per inpatient day were produced using a high-climate-impact electricity mix. The reported climate impact per litre of oxygen in our sensitivity analysis is similar to reported values^24^. However, the volume of oxygen that was attributed to each inpatient day when treating septic shock and medical ICU patients in the US and Australia was only approximately 2000 l^12–13^. No previous study has reported the contributions of various respiratory support modes to total oxygen consumption. An increased consumption of oxygen at the studied hospital is most likely due to a relatively high use of high-flow oxygen therapy. Also, differences in illness severity, with a larger fraction of patients receiving respiratory support in our study, may contribute to this disparity. Moreover, the medical ICU study from the US calculated medical gas use based on the clinical hospital floor area. This approach may underestimate actual medical gas use because oxygen use may be higher in an ICU than in the average clinical unit. We conclude that in a setting in which high-flow oxygen therapy predominates, the climate impact from oxygen is higher than previously recognised.

### Mitigation strategies

Our results suggest that the foremost opportunity to reduce the climate impact of ICUs in countries with low-climate-impact energy mixes is to reduce the use of single-use items. Based on our study, single-use aprons, gloves, woven gauze, syringes, and wash wipes accounted for 57% of the climate impact from single-use items (Fig. 2). The contribution of these products aligns with a previous study that included single-use items, cleaning products, pharmaceuticals, and textiles that were used at an ICU in the Netherlands^10^ and collectively, the results suggest that such products constitute robust targets for climate impact mitigation strategies.

Notably, a previous report indicate that nonsterile gloves are overused and misused up to 40% of the time, leading to unnecessary use of resources and potentially increasing the risk of cross-contamination^25^. Thus, educating staff about the benefits of adhering to recommendations on proper glove use is an opportunity to reduce the impacts and the risk of cross-contamination^25^. Also, the use of single-use items could be minimised by switching to reusable instruments when such alternatives are available and have similar clinical performance. For example, reusable textiles have been shown to have approximately one-sixth of the environmental impact of single-use options^5^. We are unaware of any study that has shown that single-use aprons are better than reusable aprons concerning the safety of patients or healthcare workers. This suggests that considerable reductions in climate impact could be achieved by replacing single-use aprons with reusable aprons without compromising the quality of care. Other options for mitigating the climate impact of ICUs regarding single-use items are the reduced use of infusion sets^7^ and pathology tests^8–9^.

By performing an exclusively process-based LCA, in which we included several components of ICU care that have not been considered in previous studies, we gained novel insights into the relative contributions of various elements of ICU care to the total climate impact. Previous studies excluded pharmaceuticals, fluids, and diagnostic imaging (except for X-rays) in their analyses. Our study showed that these components account for 8% of the total climate impact, which suggest that their contribution is sizable and that they represent potential targets for climate impact mitigation efforts.

The foremost opportunity for countries that are dependent on high-climate-impact energy mixes is to accelerate the transition to low-climate-impact energy mixes. Another important mitigation strategy is to reduce overall energy consumption by limiting the number of hours that electronic equipment is on standby, upgrading old equipment to newer, more energy-efficient models, and restricting the use of equipment with high energy requirements whenever possible.

### In perspective

Based on data from the Swedish intensive care registry, we deduced that the number of ICU inpatient days in Sweden in 2022 was 117,190, including all types of ICUs, with a total of 42,238 patients being treated—thus, the average patient was treated for 2.77 days^18^. Assuming that the average climate impact per inpatient day is similar for all types of ICUs in Sweden, the annual climate impact of intensive care in Sweden was 3515 MtCO_2_eq (82 kg CO_2_eq per patient), or 0.004% of the yearly national climate impact of 88 million MtCO_2_eq, in 2022^26^. When using a high-climate-impact energy mix, the climate impact was 350 kg CO_2_eq per ICU patient. In comparison, the climate impact from operating theatres in the US, UK, and Canada ranged between 146 kg and 232 kg CO_2_eq per case^27^. Thus, our data suggest that ICU care per patient has a lower climate impact than that generated per case in operating theatres when using a low-climate-impact energy mix and higher climate impact when using a high-climate-impact energy mix. However, given the larger throughput of patients in operating theatres compared with ICUs, surgical operating theatres will likely have a larger overall climate impact than intensive care.

### Limitations

A potential limitation of this study is that our model did not include oxygen that was delivered via face masks or nasal prongs. However, since the introduction of nasal high-flow devices, it has become uncommon to provide oxygen through face masks and nasal prongs for extended periods of time in the studied ICU. Their contribution to the total climate impact is likely minor compared with the oxygen that is delivered by nasal high flow. Another potential limitation of our study is the cutoff values that we used to select the single-use items, pharmaceuticals, and fluids for inclusion in the analysis. We selected items based on the number that had been procured, rather than weight. Thus, we might have overlooked heavy items with a potentially high climate impact per item, of which few were procured. Further, pathology tests that were performed in another unit were outside the system boundaries of our primary analysis. However, among elements of pathology testing, that with the highest climate impact can be traced to single-use materials, such as needle holders, blood collection tubes, gloves, and swabs^9^. Because these items were included in our analysis, the omission of pathology testing is unlikely to have impacted our results to any larger extent.

Patient characteristics and the type of care that is delivered by an ICU may differ between countries and across ICUs within a country, limiting the external validity of our results. For example, in contrast to Swedish ICUs, oral feeding may be common in some settings and contribute to the climate impact. Thus, in the previously mentioned hybrid LCA from a medical ICU in the US, patient food represented more than 10% of the total climate impact^13^. In such settings, mitigating strategies targeting energy consumption associated with food preparation as well as hospital food waste are warranted^28^. We also acknowledge that our study was performed in a high-income country. Therefore, the results may not be representative of middle-income and low-income countries, necessitating additional studies to assess hotspots that could be targeted for mitigation in such countries.

## Conclusion

Reducing the use of single-use items is the foremost opportunity to reduce the climate impact of intensive care in high-income countries with low-climate-impact energy mixes. In contrast, transitioning to renewable energy or lowering energy use is the foremost opportunity to reduce the climate impact of intensive care in countries that are dependent on high-impact energy mixes.

## Electronic supplementary material

Below is the link to the electronic supplementary material.

Supplementary Material 1

## Data Availability

All data generated or analysed during this study are included in this published article and its additional files.

## Abbreviations

API: Active pharmaceutical ingredients
CT: Computed tomography
EIO-LCA: Economic input-output life cycle assessment
GWP: Global warming potential
HVAC: Heat ventilation air conditioning
ICU: Intensive care unit
ISO: International organization for standardization
LCA: Life cycle assessment
